# Assessing 5-Aminolevulinic Acid as a Natural Biocide Precursor for Light-Activated Eradication of *Pseudomonas* spp.

**DOI:** 10.3390/ijms26157153

**Published:** 2025-07-24

**Authors:** Irena Maliszewska, Anna Zdubek

**Affiliations:** Department of Organic and Medicinal Chemistry, Faculty of Chemistry, Wrocław University of Science and Technology, Wybrzeże Wyspiańskiego 27, 50-370 Wrocław, Poland; anna.zdubek@pwr.edu.pl

**Keywords:** antimicrobial photodynamic inactivation, protoporphyrin, virulence factor

## Abstract

Photodynamic inactivation (aPDI) involves the interaction of three components: non-toxic photosensitizer molecules (PS), low-intensity visible light, and molecular oxygen. This interaction leads to the generation of toxic reactive oxygen species. The present work demonstrated the efficacy of light-induced antimicrobial photodynamic inactivation against *Pseudomonas aeruginosa* and *Pseudomonas putida* using 5-aminolevulinic acid (5-ALA) as a prodrug to produce the photosensitizer protoporphyrin IX. The photoeradication efficiency of these pathogens under blue (405 nm; 45 mW cm^−2^) and red (635 nm; 53 mW cm^−2^) light was investigated. Results showed that at least 30 min of blue light irradiation was necessary to achieve a 99.999% reduction of *P*. *aeruginosa*, whereas red light was less effective. *P. putida* exhibited limited susceptibility under similar conditions. To enhance aPDI efficiency, exogenous glucose was added alongside 5-ALA, which significantly increased the photodynamic efficacy—particularly against *P. aeruginosa*—leading to complete eradication after just 5 min of exposure. Spectroscopic analyses confirmed that glucose increased the levels of protoporphyrin IX, which correlated with enhanced photodynamic efficacy. Furthermore, multiple aPDI exposure reduced key virulence factors, including alkaline protease activity, biofilm formation, and swarming motility (in *P. aeruginosa*). These findings suggest that 5-ALA-mediated photodynamic inactivation offers a promising strategy to improve efficacy against resistant Gram-negative pathogens.

## 1. Introduction

*Pseudomonas* is one of the most extensively studied microorganisms and represents the genus with the highest number of species among Gram-negative bacteria. *Pseudomonas* spp. are strictly aerobic organisms commonly found in soil, water, and marine environments [1]. These bacteria may constitute part of the normal human flora, but they are predominantly isolated in association with nosocomial opportunistic infections [2]. The pathogenic mechanisms of *Pseudomonas*-induced disease are complex and involve the production of extracellular proteases and other toxic proteins, as well as hemolysins, endotoxins, and exotoxin A [3].

It is well known that infections caused by *Pseudomonas* spp. are particularly challenging to treat due to their intrinsic resistance to many conventional antibiotics [4]. Consequently, treatment of infections caused by these pathogens generally requires the administration of two or three antimicrobial agents or the use of antibiotics that are infrequently utilized in routine clinical practice, such as polymyxins, including colistin. Therefore, it is justified to take an interdisciplinary approach to combating infections caused by strains of the *Pseudomonas* genus and to supplement traditional antibiotic therapy with antibacterial photodynamic inactivation (aPDI), which represents a promising method for treating infections caused by Gram-negative pathogens [5].

Photodynamic inactivation utilizes non-toxic molecules known as a photosensitizer (PS) along with low-intensity visible light. When combined with oxygen, this interaction generates toxic reactive species [6]. One of aPDI’s key benefits is its dual selectivity: the photosensitizer can be directed specifically to the target cells or tissues, and the light can be precisely focused on the affected area.

Photodynamic inactivation has been proven to be effective in the killing of bacteria from the genus *Pseudomonas*, and several approaches to light-induced eradication of these pathogens have been described to date. For example, the effectiveness of aPDI utilizing methylene blue (MB) against planktonic *P. aeruginosa* was evaluated at various MB concentrations and different light exposure parameters to identify the most effective combination for bacterial inactivation. The obtained results indicated the greatest reduction in bacterial viability—corresponding to a 3.48 and 4.32 log reduction—at light doses of 90 and 108 J cm^−2^, respectively, with MB concentrations of 500 μg mL^−1^ and 250 μg mL^−1^ [7]. In another study, the combined effect of curcumin as a photosensitizer with polymyxin B on *P. aeruginosa* was evaluated, revealing that the synergistic action of curcumin and polymyxin B effectively killed these bacteria [8]. An in vitro study assessed the efficacy of chlorin e6-PDI in combating suspensions of multiple bacterial species, including *P. aeruginosa*, demonstrating a significant reduction in viable bacteria following treatment with chlorin e6 at a concentration of 10 μM and a light dose of 20 J cm^−2^ [9].

An alternative approach involves the combination of inorganic salts with photosensitizers. Michael Hamblin investigated the effects of selected inorganic salts—such as sodium azide, potassium iodide, potassium bromide, and thiocyanate—in conjunction with antimicrobial photodynamic inactivation [10]. A significant enhancement was demonstrated in the antimicrobial efficacy of these compounds. However, the authors also noted certain limitations, including toxicity, reduced oxidation capacity, and moderate effectiveness. Ultimately, the findings indicated that among the tested salts, potassium iodide was the most effective and non-toxic to mammalian cells at concentrations up to 100 mM [11]. Similar studies were conducted by Vecchio et al. [12]. The researchers employed aPDI in combination with potassium iodide (KI), demonstrating a synergistic antimicrobial effect both in vitro and in vivo on bacterial cultures.

The identification that 5-aminolevulinic acid (5-ALA) can serve as a natural precursor to the photosensitizer has facilitated numerous applications wherein this amino acid or its ester derivatives are regarded as prodrugs requiring metabolic conversion into endogenous porphyrins-like compounds to attain their active photosensitizing properties [13]. The Food and Drug Administration has approved 5-ALA, in conjunction with light irradiation, for the treatment of certain carcinomas [14]. Achieving an adequate photosensitizer concentration for effective lesion destruction necessitates exogenous administration of 5-ALA, and, consequently, in this therapy, 5-ALA is applied topically in the form of a cream or gel approximately 3–5 h prior to illumination. Additionally, 5-ALA can be administered orally, via infusion, or through inhalation routes. This amino acid exhibits several advantages over other photosensitizers. Due to its natural origin, it is biocompatible and minimizes adverse reactions, which translates into reduced toxicity and a lower risk of side effects. It is considered that the price of this drug is also satisfactory.

One significant area of research pertains to the role of glucose in photodynamic therapy using 5-ALA as a precursor of the intracellular photosensitizer. Given that most studies utilizing this amino acid have focused on the effective photoinactivation of tumors, the majority of existing research findings relate to this group of pathogenic cells. It is well established that tumor cells exhibit higher glucose consumption compared to normal cells and, consequently, this preferential uptake has been exploited in light-induced therapy through the use of photosensitizer conjugates with glucose, facilitating targeted delivery to tumor-affected sites. For example, the synergistic effect of administering glucose in conjunction with 5-ALA in tumor inactivation was investigated by Nelson et al. [15], and the results obtained demonstrated that glucose, when combined with PDT, led to a higher proportion of cured animals compared to the use of PDT alone. Furthermore, the findings indicated that this approach required a lower light dose and could potentially allow for a reduction in the photosensitizer dosage, thereby achieving optimal therapeutic response with minimal side effects in cancer treatment.

Ambiguous data are available regarding the antibacterial efficacy of photodynamic inactivation utilizing 5-aminolevulinic acid (5-ALA) against *P. aeruginosa* [16,17,18,19], and to the best of our knowledge, there are no such data pertaining to *P. putida*. Furthermore, the role of glucose in the light-induced inactivation of prokaryotic cells mediated by this amino acid as a prodrug has not been previously demonstrated.

This study evaluated the application of 5-ALA in antimicrobial photodynamic inactivation targeting two clinically significant pathogens: *P. aeruginosa* and *P. putida*. An effective strategy to enhance the light-mediated inactivation of these bacteria was proposed, involving the exogenous administration of glucose in conjunction with this amino acid. The impact of multiple photodynamic inactivation treatments on the reduction in certain virulence factors and the emergence of tolerance to aPDI in these pathogens was also examined.

## 2. Results

**Figure 1:**
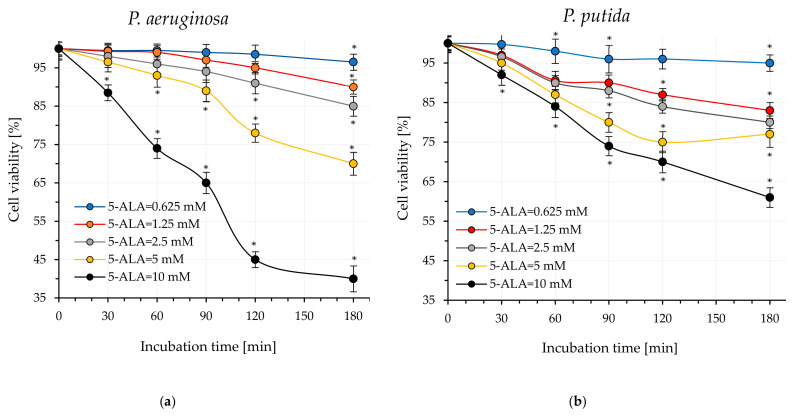

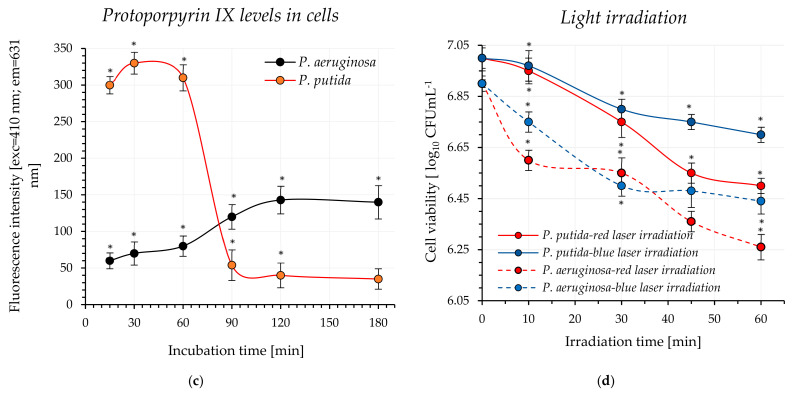
Effect of 5-ALA concentration on the viability (in darkness) of (**a**) *P. aeruginosa*; (**b**) *P. putida*. Effect of incubation time with 5-ALA on the fluorescence intensity of protoporphyrin IX accumulated in cells (without exposure of bacteria to light) (**c**). Effect of irradiation time on bacterial cell viability (in the absence of exogenous 5-ALA) (**d**). Data presented as the arithmetic mean (±SD) from two independent experiments. Asterisks indicate the statistical differences *p* < 0.05.

Our experiments on the light-induced inactivation of *P. aeruginosa* and *P. putida* were preceded by determining the non-cytotoxic concentration of 5-ALA in darkness, as well as the optimal external incubation time with this amino acid to achieve the highest intracellular concentration of protoporphyrin IX. Additionally, it was important to estimate cell viability by assessing the phototoxic effect of endogenous photosensitizers after irradiation of the pathogens with blue and red laser light. The results of these investigations are summarized in Figure 1a–d.

### 2.1. Effect of 5-ALA Concentration on Bacterial Viability (Dark Cytotoxicity)

Analysis of the effect of 5-ALA on bacterial cells demonstrated that this amino acid influences bacterial viability in darkness. The mortality rate was dependent on both the concentration of 5-ALA and the incubation time with bacteria. The highest observed mortality (regardless of bacterial species) occurred when 5-ALA was applied at a concentration of 10 mM, resulting in approximately a 40 ± 2% reduction in the number of viable cells after 180 min of incubation (Figure 1a,b). It was assumed that the highest concentration of 5-ALA that caused bacterial mortality in the dark not exceeding 20% should be used in photodynamic inactivation of pathogens. For both bacterial strains studied, this criterion was met by a 5-ALA concentration of 2.5 mM.

### 2.2. Effect of the Time of Administration of Extracellular 5-ALA on the Accumulation of Protoporphyrin IX

The key factor affecting the effectiveness of light-induced killing of *P. aeruginosa* and *P. putida* was the time of bacterial incubation with external 5-ALA in the dark (pre-irradiation time), which led to the highest levels of protoporphyrin IX within the cells. Therefore the effect of time of incubation of bacterial cells with 5-ALA (at non-cytotoxic concentration) on the level of protoporphyrin IX accumulated in the bacteria was examined, and the results obtained are shown in Figure 1c. It was assumed that changes in fluorescence intensity at 631 nm corresponded to alterations in the accumulated level of protoporphyrin IX within the bacterial cells. As can be seen in Figure 1c, the fluorescence intensity of protoporphyrin IX depended on the incubation time of bacterial cells with 5-ALA. In the case of the *P. aeruginosa* lysate, the fluorescence intensity increased up to 120 min of incubation, reaching a maximum value. After 180 min of incubation, the protoporphyrin levels in these bacterial cells slightly decreased. For *P. putida*, an increase in fluorescence intensity was observed within the first 15–30 min. From the 60th minute of incubation, a marked decrease in the level of protoporphyrin IX in cells was clearly observed. Consequently, all subsequent experiments involved pre-incubating *P. aeruginosa* with 5-ALA for 120 min and *P. putida* with 5-ALA for 30 min prior to irradiation (pre-irradiation time).

### 2.3. Impact of Light Exposure Time on Bacterial Viability

In this experiment, pathogenic cells were exposed to blue (410 nm) and red light (635 nm) for 60 min (in the absence of exogenous 5-ALA). The number of viable cells in suspensions was analyzed after 10, 30, 45, and 60 min of light exposure (Figure 1d). Based on the conducted experiments, the following conclusions were drawn: firstly, red light was more bactericidal regardless of the bacterial species, and the biocidal effect was dependent on the light dose (exposure time). Secondly, *P. aeruginosa* appears to be more sensitive to blue/red light compared to *P. putida*. After 60 min of exposure to blue light, the number of viable cells of this microorganism decreases by 0.46 ± 0.03 log units, corresponding to a mortality rate of approximately 66.4%. When red light was used in the experiment, the reduction in viable bacteria was 0.64 ± 0.05 log units, correlating with a mortality rate of approximately 77.1%. Under identical experiment conditions, the reduction in viable *P. putida* was 0.3 ± 0.03 log units for blue light and 0.5 ± 0.03 log units for red light. These values correlated with cell mortality rates of approximately 49.9% for blue light and 50.1% for red light.

### 2.4. Photodynamic Inactivation of Bacteria

**Figure 2:**
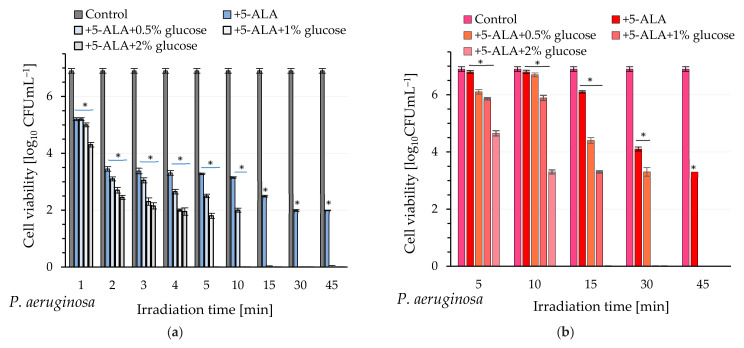

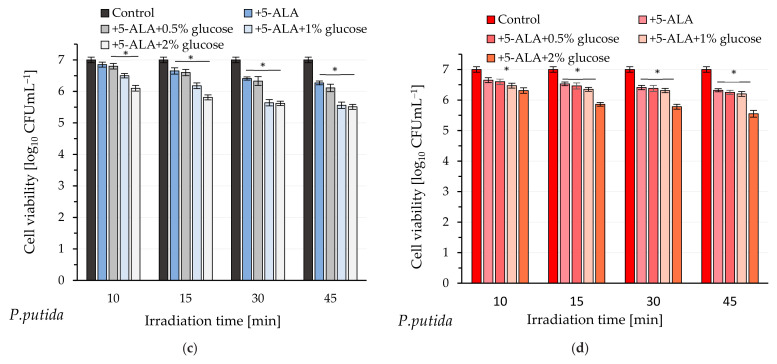
Efficiency of photodynamic inactivation of *P. aeruginosa* and *P. putida* under various experimental conditions. (**a**,**c**) Blue light (410 nm); (**b**,**d**) red light (635 nm). Data presented as the arithmetic mean (±SD) from two independent experiments. Asterisks indicate the statistical differences with *p* < 0.05.

The next step of our study involved exposing bacteria to blue and red light following pre-incubation with 5-ALA at a non-toxic concentration (2.5 mM; in darkness) for 120 or 30 min, respectively, for *P. aeruginosa* and *P. putida*. The survival rates of these bacteria are summarized in Figure 2a–d. As anticipated, bacterial lethality depended on the type of laser used (light wavelength), and regardless of the tested microorganism, blue light exhibited a greater biocidal activity.

Cell mortality after blue light exposure was correlated with the duration of light exposure, i.e., the light dose. Notably, a significant reduction in viability of *P. aeruginosa*, reaching a log reduction of 3.75 ± 0.03 (corresponding to a 99.982% kill rate), was observed after 10 min of blue illumination. Extending the exposure time to blue light to 15 min resulted in a bacterial kill level of 4.40 ± 0.03 log units, corresponding to a 99.996% reduction in viable bacteria. When the irradiation was 30–45 min, cell mortality increased to 99.999%, corresponding to a reduction in cell number by 4.9 ± 0.03 logarithmic units (Figure 2a).

Red light (635 nm) demonstrated lower biocidal efficacy, with significant cell mortality observed after 30–45 min of irradiation. The reduction in viable bacterial counts was 2.80 ± 0.04 and 3.60 ± 0.07 log units, corresponding to pathogen mortality levels of approximately 99.840% and 99.975%, respectively (Figure 2b).

The photoinactivation efficiency of *P. putida* in comparison to *P. aeruginosa* was significantly lower, regardless of the laser used. It was observed that, following irradiation of this pathogen with blue light for 45 min (after an initial incubation with 5-ALA for 30 min), approximately 81.5% of the bacteria were eliminated, corresponding to a logarithmic reduction of 0.73 ± 0.06. Shorter exposure times (30 min) to blue light resulted in the inactivation of no more than 74% of the bacterial population (reduction bacterial cells by 0.59 ± 0.05% log_10_ unit) (Figure 2c).

The efficacy of light-induced inactivation of *P. putida* using red light was also lower compared to blue light. It was demonstrated that a 45 min exposure resulted in a bacterial mortality rate of approximately 80.0%, corresponding to a reduction of 0.68 ± 0.05 log units in bacterial count. Shorter irradiation durations were, of course, less effective; specifically, a 30 min exposure under identical experimental conditions achieved a bacterial mortality rate of approximately 75.0% (Figure 2d).

**Figure 3:**
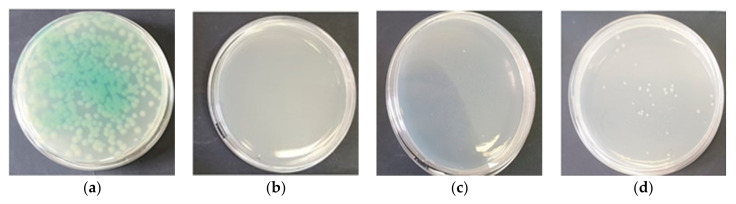
Growth of P. aeruginosa on Mueller–Hinton agar after irradiation with blue light: (**a**) for 5 min in the presence of 5-ALA; (**b**) for 5 min in the presence of 5-ALA and 2% glucose; (**c**) for 10 min in the presence of 5-ALA and 1% glucose; (**d**) for 10 min in the presence of 5-ALA and 0.5% glucose.

In the next stage of our experiments, an increase in light-induced bacterial mortality was observed after administration of 5-ALA and glucose to the cells. The obtained results are summarized in Figure 2a–d. It was clearly demonstrated that the efficiency of photoinactivation of the studied pathogens in the presence of glucose depended on the concentration of this monosaccharide in the mixture and, of course, on the wavelength used. For example, an extremely short irradiation time of *P. aeruginosa* (1 min; blue light) resulted in a reduction in viable cell counts by 1.7 ± 0.05, 1.9 ± 0.07 and 2.60 ± 0.08 log units in the presence of 5-ALA and glucose at concentrations of 0.5%, 1.0%, and 2.0%, respectively (Figure 2a). A lethal effect (viable cell count below the detection level) was achieved after 5 min of exposure to blue light in the presence of 5-ALA and 2% glucose. When glucose was used at concentrations of 1% and 0.5%, the lethal effect on this pathogen was observed after 10 and 15 min of irradiation, respectively (Figure 2a and Figure 3).

On the other hand, after 5 min of exposure of these bacteria to red light, the reduction in viable *P. aeruginosa* was 0.80 ± 0.08, 1.04 ± 0.04, and 2.25 ± 0.09 log units, corresponding to pathogen mortality rates of 84.8%, 90.9%, and 99.4%, respectively, when glucose was applied at concentrations of 0.5%, 1%, and 2%. Extending the irradiation time to 15 min (under the same experimental conditions) resulted in a lethal effect (viable bacterial count below the detection limit) when glucose was present at a concentration of 2%. The lethal effects following exposure of bacteria to red light for 30 min in the presence of 5-ALA and 1% glucose were also observed. Achieving a lethal effect in the presence of 0.5% glucose required a 45 min exposure of these bacteria to red light (Figure 2b). The lethal effect was not achieved in the case of *P. putida*, even in the presence of 2% glucose (Figure 2c,d).

**Figure 4:**
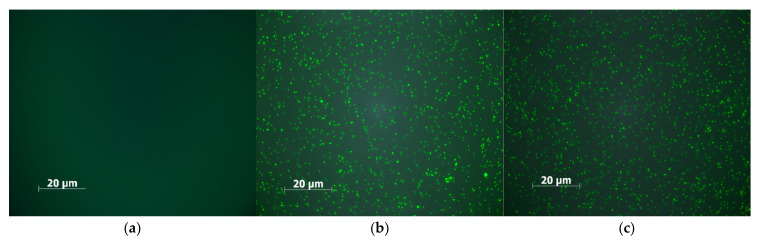
Fluorescence microscopy images of *P. fluorescens* before and after blue light exposure. Non-viable bacteria appear as green dots. (**a**) Cells before light treatment; (**b**) cells after 15 min of light exposure in the presence of 5-ALA alone; (**c**) cells after 1 min of light exposure in the presence of 5-ALA and glucose at a concentration of 2%.

Fluorescence microscopy was employed to assess the bactericidal efficacy of 5-ALA-mediated antimicrobial photodynamic inactivation and to demonstrate the influence of glucose on the effectiveness of this approach. This high-affinity nucleic acid dye selectively enters cells with damaged plasma membranes while remaining excluded from intact cells. Representative images of *P. aeruginosa* cells before and after 10 min of blue light irradiation in the presence of 5-ALA alone and 5-ALA + glucose at a concentration of 2% are shown in Figure 4. Non-viable bacteria are labeled by SYTOX Green and appear as green fluorescent dots. The intensity of green fluorescence correlates with the degree of bacterial cell death.

The number of green dots (indicating dead bacteria) is similar in images in Figure 4b,c, demonstrating that a very high efficiency of bacterial photoinactivation was achieved at energy doses of approximately 45 J cm^−2^ and 3 J cm^−2^ (corresponding to 45 min and 1 min of blue irradiation, respectively). Reducing the light dose to achieve efficient antimicrobial photodynamic inactivation was possible through the delivery of glucose to the cells in addition to 5-ALA.

A comparable enhancement in photodynamic efficiency resulting from exogenous glucose supplementation was also observed in *P. putida* cells. The highest mortality was achieved by adding 2% glucose to the mixture, followed by a 45 min light irradiation (regardless of the light used). When bacteria were exposed to blue light, the number of viable cells decreased by 1.49 ± 0.08 logarithmic units, corresponding to a mortality rate of approximately 96.8% (Figure 2c). Under identical experimental conditions, but with red light exposure, the number of viable cells decreased by 1.45 ± 0.11 logarithmic units as well, which corresponds to a mortality rate of approximately 96.4% (Figure 2d).

### 2.5. Effect of Glucose on Intracellular Photosensitizer Levels

**Figure 5:**
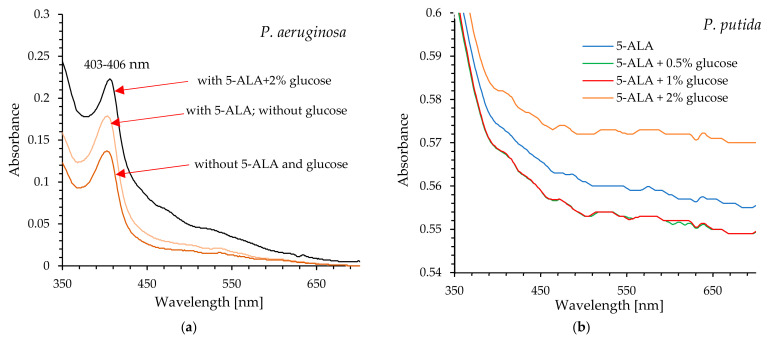

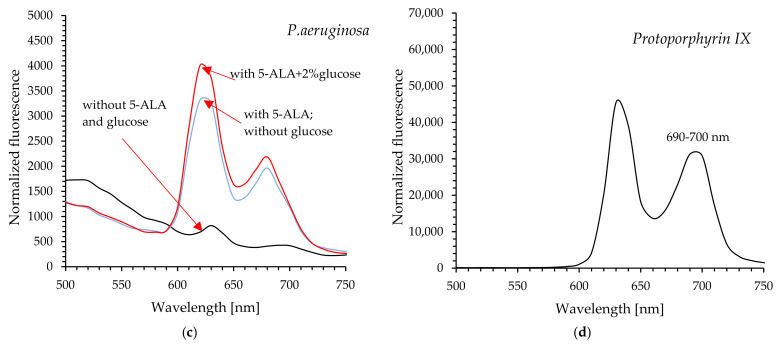
UV-Vis absorption spectra of cell-free lysates (**a**,**b**); fluorescence spectra of cell-free lysates (**c**); fluorescence spectrum of protoporphyrin IX in lysis buffer (Tris-acetate-EDTA with 2% SDS) (**d**).

To validate the effect of glucose on the level of endogenous photosensitizers in bacterial cells, UV-Vis spectra and fluorescence spectra of cell-free lysates of the tested bacteria were recorded. The UV-Vis spectra shown in Figure 5a are characteristic for lysates from *P. aeruginosa*. These spectra exhibit a distinct absorption maximum at a wavelength of 403–406 nm, the Soret band described for protoporphyrin IX (see Figure S1; Supplementary Information).

The absorbance value at this wavelength varies depending on the conditions under which the cells were incubated prior to lysis, and the highest absorbance was observed for the lysate derived from cells incubated with 5-ALA in the presence of glucose. On the other hand, UV-Vis spectra obtained for lysates from *P. putida* did not show clear absorption maxima in the range of 380-700 nm (Figure 5b). The UV-Vis spectrum of 5-ALA in water is shown in Figure S2 (Supplementary Information). As shown in this figure, the studied 5-ALA is characterized by a maximum absorption in the range of 265–275 nm.

Analysis of fluorescence spectra (Figure 5c) showed that cell-free lysates from *P. aeruginosa* exhibited two emission maxima at wavelengths of 620–630 nm and 680–700 nm. The fluorescence intensity was the lowest in lysates obtained from these bacteria without exogenous 5-ALA (control lysate), with the fluorescence maxima at 630 nm and 700 nm. Delivery of exogenous 5-ALA to cells resulted in nearly a 4-fold increase in fluorescence intensity and a shift in the fluorescence maximum to 620 nm and 680 nm. The highest fluorescence intensity was observed in the lysate from bacteria incubated with 5-ALA in the presence of glucose, representing almost a 5-fold increase. The fluorescence maxima remained at 620 nm and 680 nm. The described fluorescence spectra are consistent with those obtained for protoporphyrin IX dissolved in the lysis buffer (Tris-acetate-EDTA with 2% SDS) (Figure 5d).

### 2.6. Effect of Multiple 5-ALA-aPDI on Bacterial Virulence Factors

The cells of the studied pathogens were subjected to five consecutive exposures to blue and red light, with each irradiation preceded by pre-incubation of the bacteria with 5-ALA alone or in combination with 2% glucose. The effects of blue light irradiation on the selected virulence factors were analyzed, and the results obtained are presented in Table 1.

As demonstrated in the above table, fivefold treatment of bacteria with blue light following incubation with 5-ALA and/or in the presence of glucose significantly affected the efficiency of biofilm formation by both studied pathogens. It was observed that *P. aeruginosa* subjected to five cycles of photoinactivation produced biofilms with approximately 40–50% reduced biomass compared to bacteria that were not exposed to light. A similar trend was noted for *P. putida*, although the reduction in biofilm production efficiency was approximately 20–40% relative to untreated controls. Additionally, significant decreases were observed in the activity of alkaline protease. The reduction in the activity of this enzyme produced by *P. aeruginosa* ranged from 27% to 35% depending on the experimental conditions. In the case of *P. putida*, the reduction in the alkaline protease activity was slightly lower compared to *P. aeruginosa* and ranged from 12% to 16%.

It was also found that the swarming motility of *P. aeruginosa* was restricted after multiple treatments with blue light in the presence of 5-ALA or 5-ALA combined with 2% glucose. Prior to multiple-light-induced inactivation, the swarming zone diameter measured approximately 6.0 ± 0.2 cm; however, after successive blue light exposures, the diameter did not exceed 4.8 ± 0.2 cm (Table S1; Supplementary Information).

The results of experiments conducted under the same conditions, but with red light used for irradiation, are presented in Table S2 (Supplementary Information). Multiple treatments of the studied pathogens with red light was less effective in reducing selected virulence factors. A significant reduction in biofilm formation efficiency on an abiotic surface was observed in both strains, amounting to no more than 10–15%. Swarm zones formed by *P. aeruginosa* after multiple exposures to red light were also smaller (by approximately 10%) compared to those of bacteria not subjected to five cycles of aPDI.

### 2.7. Effect of Multiple Exposures to Light on Tolerance to aPDI

The studied bacteria were exposed to five cycles of blue light in the presence of exogenous 5-ALA, as well as 5-ALA combined with glucose according to the method described in Section 4.8. Following these treatments, the resulting cultures were exposed to light under conditions described in Section 4.5, and their susceptibility to antimicrobial photodynamic inactivation was assessed. The obtained results are summarized in Figures S3a–d (Supplementary Information). As shown in these figures, red light was less effective compared to blue light, and *P. aeruginosa* exhibited greater sensitivity to the treatment compared to *P. putida*. Moreover, it was shown that, in most cases, an increased cell mortality was observed compared to bacteria that were not exposed to multiple light treatments. For example, after 30 min of red light treatment in the presence of 5-ALA, the number of viable cells of *P. aeruginosa* decreased by 3.20 ± 0.09 log units (compared to 2.80 ± 0.04 log units). A similar effect of increased mortality of *P. putida* was observed after 15 min of red light irradiation in the presence of 5-ALA and glucose, and the number of viable cells decreased by 1.65 ± 0.07 log units (compared to 1.14 ± 0.03 log units). No reduction in sensitivity to light-induced bacterial cell mortality was observed under the described experimental conditions.

## 3. Discussion

**Figure 6:**
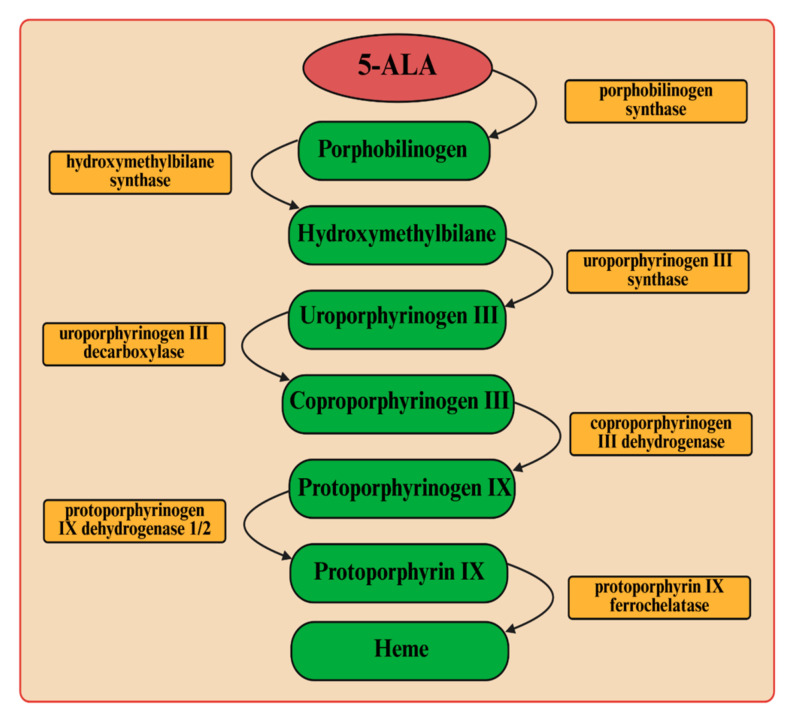
Metabolic pathway of 5-aminolevulinic acid to heme.

In these studies, two Gram-negative bacterial species were used: *Pseudomonas aeruginosa* and *Pseudomonas putida*. To combat these pathogenic bacteria, we employed light-induced inactivation (aPDI), a technique with a longstanding history that has garnered renewed interest in recent years owing to the significant increase in bacterial antibiotic resistance. The endogenous production of porphyrin-like compounds from 5-ALA was used as a tool to enable bacterial control (Figure 6). These molecules act as photosensitizers and, upon excitation, interact with molecular oxygen (in its triplet state (^3^O_2_)), which is the predominant form of oxygen in biological systems. The energy transfer from the excited triplet state of photosensitizer to molecular oxygen results in the formation of singlet oxygen (^1^O_2_), a highly reactive molecule [14,20]. Singlet oxygen reacts with various biological substrates, leading to oxidative damage of cellular biomolecules, including lipids, proteins, and nucleic acids [21]. It is important to note that singlet oxygen has a very limited diffusion radius—approximately 0.02 μm—and a short lifetime within biological environments. These characteristics enable its use for localized therapeutic effects, minimizing damage to surrounding healthy cells and tissues [22].

In our experiments, light at wavelengths of 405 nm and 635 nm was used for photodynamic destruction of *P. aeruginosa* and *P. putida*. It is assumed that, in the case of Gram-negative bacteria, intracellular protoporphyrin IX is responsible for cell photosensitization using visible light [13]. Protoporphyrin IX is an endogenous fluorescent heterocyclic organic molecule that occurs in all living cells. This molecule is an essential precursor with specific physiological functions in bacteria, such as oxygen transport and storage via heme (iron/zinc-PPIX). It is also a ubiquitous prosthetic group of proteins such as cytochrome, catalases, peroxidases, and nitrate reductase. It was previously shown that the UV–visible absorption spectra of protoporphyrin IX are characterized by a prominent Soret band and four weaker Q bands (QI, QII, QIII, and QIV) [23]. The Soret band is centered around 406 nm, while the Q bands are observed in the range of approximately 532–641 nm (their exact positions depend on the solvent used).

The results obtained showed that red light (without the addition of an exogenous photosensitizer) induced bacterial cell death, with the efficacy depending on the microorganism and the light dose. The bacterial mortality rate ranged from approximately 55% to 77%, with *P. aeruginosa* exhibiting greater sensitivity to red light.

A blue light was also effective in destroying the studied pathogens, which was not an unexpected finding. It is well known that blue light (aBL) within the wavelength range of 400–470 nm caused inactivation of bacteria, and this antimicrobial approach, particularly in the context of skin infections, is being increasingly explored [24]. Numerous studies have confirmed the efficacy of aBL in bacterial eradication but the precise mechanism of BL action remains unclear. It is widely accepted that aBL stimulates endogenous porphyrin-like compounds and activates the production cascade of reactive oxygen species (ROS). These toxic ROS interact with various bacterial cellular components, ultimately leading to cell lysis.

Our study primarily focused on evaluating the efficacy of pathogen eradication using laser blue and red light, employing 5-ALA as an exogenous precursor of the active “biocide”(protoporphyrin IX). It was determined that achieving the targeted mortality rate of *P. aeruginosa* (99.999%) required a minimum of 30 min of exposure to blue light, corresponding to a light dose of 81 J cm^−2^. In the case of red light, even 45 min of irradiation, delivering a dose of 143 J cm^−2^, did not result in the desired eradication effect. One possible reason for the limited inactivation effectiveness is the weak absorption of PpIX in the red spectral region compared to its absorption near the Soret band. This assumption was previously confirmed in *Propionibacterium acnes* (*Cutibacterium acnes*), which accumulates high levels of PpIX [25], but inactivation of this pathogen with red light required high light doses to achieve a significant decrease in viability [26]. Similar results were also observed with another Gram-negative bacterium, *E. coli* [27]. Hsieh et al. [18] observed a significant decrease in bacterial viability for *P. aeruginosa*, where 5.0 mM 5-ALA was photosensitized by accumulating a red light dose of 162 J cm^−2^.

Analysis of the photoinactivation efficiency of *P. aeruginosa* demonstrated by other researchers has shown that aPDI based on 5-ALA is an effective method for combating these pathogens. However, it requires relatively high concentrations of this amino acid as a precursor to the active photosensitizer and substantial light doses. For example, Lee et al. [16] reported that pre-incubation of *P. aeruginosa* with 5-ALA at a concentration of 20 mM combined with red light irradiation at 630 nm (light dose of 240 J cm^−2^) resulted in lethal photoinactivation. Similarly, using 7.5 mM 5-ALA with a light dose of 360 J cm^−2^ also achieved effective inactivation.

In a different study [19], the authors demonstrated that effective inactivation of *P*. *aeruginosa* via visible light-induced photoinactivation (using a halogen-tungsten lamp) required a 5-aminolevulinic acid (5-ALA) concentration of 40 mM and a light dose of 142 J cm^−2^.

When *P. putida* was used in our experiments, it was observed that, regardless of exposure duration up to 45 min to either blue or red light (light dose of 121 J cm^−2^ and 143 J cm^−2^, respectively), the mortality rate did not reach the 99.999% threshold. To the best of our knowledge, no research results have been published to date regarding the use of 5-ALA in the photodynamic eradication of *P. putida*; therefore, the data obtained cannot be compared with the results presented by other authors. This is likely due to the fact that this microorganism has not been identified as a medical concern.

Inspired by our previous discoveries [28], this study proposed a simple method to enhance the efficiency of aPDI, involving the exogenous delivery of not only 5-ALA but also glucose to the cells. The light-induced mortality of the examined pathogens, which depended on the glucose concentration and the duration of light exposure, was studied. It was demonstrated that incubation of *P. aeruginosa* cells with 5-ALA in the presence of 2% glucose and exposing these bacteria to blue light resulted in a lethal effect (visible cell count below the detection level) after just 5 min (light dose of 13.5 J cm^−2^). Lower glucose concentrations (1% and 0.5%) also induced a lethal effect, but the required light dose was in the range of 27–40 J cm^−2^. A similar phenomenon was observed with red light, but achieving a lethal effect required prolonged exposure times of 15 and 30 min for glucose concentrations of 1% and 0.5%, respectively (light dose of 48–95 J cm^−2^).

The acquisition of cell-free lysates and the analysis of their UV-Vis and fluorescence spectra clearly showed that glucose increased the level of protoporphyrin IX in *P. aeruginosa* cells, and consequently, enhanced photodynamic inactivation of this pathogen was possible after exposure to light. It seems obvious that excitation of protoporphyrin IX at the Soret band maximum results in high efficacy of bacterial killing by blue light (compared to red light).

*P. putida* cells exhibited reduced sensitivity to light even in the presence of glucose. Although the efficiency of photo-destruction was higher in the presence of this monosaccharide, a lethal effect was not observed within 45 min of exposure to blue or red light. Exogenous administration of 5-ALA and glucose at a concentration of 2% resulted in approximately 96.8% bacterial mortality; however, this outcome remains unsatisfactory. The UV-Vis spectral analysis of cell-free lysates obtained from this pathogen does not definitively confirm the presence of absorption maxima characteristic of protoporphyrin IX. It appears that minor absorption maxima are observable near 410 nm and 630 nm, particularly in the spectrum of bacterial lysate obtained from cells supplemented with 5-ALA and 2% glucose (these findings are not definitive). Additionally, the lysates did not exhibit emission spectra characteristic of protoporphyrin IX (Figure S3; Supplementary Information). It is believed that the formation of photodynamically inactive porphyrinogens (e.g., coproporphyrinogen, uroporphyrinogen), together with the weak accumulation of photodynamically active PpIX, may be responsible for the weak photoinactivation of *P. putida*.

The dependence of aPDI efficiency based on 5-ALA has been the subject of previous analyses. For example, Fotinos et al. [17] demonstrated a strong correlation between aPDI efficiency and the total amount of endogenous porphyrins produced in *E. coli* K-12, whereas no such correlation was observed in other species (*S. aureus* and *P. aeruginosa*). It is believed that this phenomenon results from differences in the types of porphyrins produced and the protective mechanisms and cellular localization of porphyrins [29]. On the other hand, a high level of photoinactivation (4-log reduction) was achieved in *P. aeruginosa* but minimal concentrations of porphyrins were detected within the cells [16]. The authors suggested that this may be due to the presence of aggregated, non-fluorescent porphyrins or porphyrinogens, which are activated during the illumination process. It has been reported that *P. aeruginosa* exposed to 5-ALA generates predominantly photodynamically inactive porphyrinogens [30].

Based on observations reported by other authors and the obtained results, it was suggested that the response to aPDI is distinctly strain-dependent in both Gram-positive and Gram-negative bacteria. Factors such as membrane composition, intracellular levels of proto- and other porphyrins, and protective mechanisms contribute to bacterial susceptibility to light-induced eradication [31].

Our previous studies confirmed that multiple sublethal and lethal doses of light, in the presence of methylene blue as a photosensitizer, slightly increased *A. baumannii*’s sensitivity to phototherapy and reduced its ability to form biofilm [32]. It has also been demonstrated that multiple photooxidations directly affect the bacteria metabolome, leading to modifications in many metabolic pathways.

This study assessed the effect of multiple photodynamic treatments on several key virulence factors of the examined bacteria. Virulence factors, especially of *P. aeruginosa*, have been extensively studied and are typically classified into three main categories: bacterial surface structures, secreted factors, and bacterial cell interactions [3]. In this work, the effect of multiple photodynamic inactivation of bacteria on the activity of alkaline protease and elastase—both classified as secreted factors—was investigated. Alkaline protease is secreted via the type I secretion system and regulated by the quorum sensing circuit [33] and can degrade complement components as well as IFN-γ and TNF-α, thereby counteracting host immune defenses and exacerbating infections [34]. Elastase destroys elastin, an essential component of lung tissue and blood vessels [35].

Lipase A, a major extracellular lipase classified as a toxin, is secreted through the type II secretion system [36]. It can damage lung tissue by degrading the major lung surfactant, dipalmitoylphosphatidylcholine, as well as host cell membranes [37]. Furthermore, studies have shown that this enzyme interacts with alginate in the biofilm matrix via electrostatic interactions, contributing to bacterial drug resistance [38]. Phospholipase C is also a toxin capable of inducing host vascular permeability and damaging organs [38].

Among the four important enzymes listed above, a significant decrease in activity due to multiple exposure to blue light was observed only for alkaline protease. The activities measured in supernatants obtained from cells incubated with 5-ALA and 2% glucose were reduced by approximately 35% and 16% (compared to pathogens not exposed to 5-ALA and light) for *P. aeruginosa* and *P. putida*, respectively.

Both examined microorganisms also exhibited a reduction in their biofilm-forming capacity on abiotic surfaces by 37–48% depending on the species and light exposure conditions. The ability to form biofilm is classified under the category of “bacterial cell interactions”. Biofilms produced by *Pseudomonas* play a crucial role in protecting the bacteria from environmental stresses and antimicrobial agents. They facilitate the bacteria’s attachment to surfaces, enabling persistent colonization in various environments, including medical devices and water systems. Within the biofilm, *Pseudomonas* bacteria communicate through quorum sensing (QS), coordinating their activities for survival and virulence. Overall, biofilm formation enhances the bacteria’s ability to persist, resist treatment, and cause chronic infections [39]. It is believed that secretion of various QS-controlled extracellular enzymes (esterases, lipases, and elastases) affects EPS (Extracellular Polymeric Substances) composition and cell motility, thereby influencing the formation and architecture of mucoid *Pseudomonas* biofilms [40]. The results of our study did not confirm correlation between lipase and phospholipase activity and the administered treatment. After five cycles of phototherapy, no significant changes were observed in the activity of these enzymes. However, a reduction in the ability of pathogens to form biofilms was noted, which may suggest a potential therapeutic effect of this method in limiting biofilm formation as a bacterial resistance mechanism.

A reduction in the swarming zone diameter detected in *P. aeruginosa* was also observed, and this phenomenon may be connected with a decrease in the ability of this bacterium to colonize diverse environments, attach to surfaces, and form biofilms. Swarming generally refers to the coordinated, collective motility of a dense bacterial population [41].

In summary, the results of these studies confirm the potential of 5-ALA as an effective prodrug in photodynamic therapy against *Pseudomonas* spp. (particularly *P. aeruginosa*), which may contribute to the development of novel therapeutic approaches. However, further research is necessary to optimize treatment parameters and enhance efficacy and safety, both of which are critical for combating resistant bacterial strains and safeguarding public health.

## 4. Materials and Methods

### 4.1. Microorganisms and Culture Conditions

In our experiments, two bacterial species served as test microorganisms: *Pseudomonas aeruginosa* ATCC 13525 and *Pseudomonas putida* ATCC 49128. A single colony of each strain was obtained from an agar plate (Mueller–Hinton Agar, BTL, Łódź, Poland) and inoculated into 5 mL of Mueller–Hinton Broth (BTL, Łódź, Poland). The resulting suspensions were incubated overnight at 37 °C and 30 °C for *P. aeruginosa* and *P. putida*, respectively. After incubation, 1 mL of each suspension was centrifuged separately for 4 min at 6000 rpm; the supernatant was discarded, and the pellet was resuspended in 1 mL of sterile deionized water. The bacterial suspensions were then adjusted to a McFarland 0.5 standard (optical density at 550 nm between 0.09 and 0.1). It was estimated that the inocula contained approximately 1 × 10^−8^ or 8 × 10^−7^ colony-forming units (CFU mL^−1^) for *P. aeruginosa* and *P. putida*, respectively.

### 4.2. Light Source

Diode lasers with the peak-power wavelength of ʎ = 405 nm (output power of 17 mW; radiation intensity of 45 mW cm^−2^) and ʎ = 635 nm (output power of 20 mW; radiation intensity of 53 mW cm^−2^) were used in this study.

### 4.3. Estimation of Dark Cytotoxicity of 5-ALA

An amount of 40 µL of 5-ALA was added to the test tubes containing 450 µL of Mueller–Hinton Broth to achieve final concentrations of 10 mM, 5 mM, 2.5 mM, 1.25 mM, 1 mM, and 0.625 mM. All these samples were then inoculated with 10 µL of the standardized bacteria suspension prepared according to the procedure described in Section 4.1. In the control sample, 5-ALA was replaced with Mueller–Hinton Broth. Subsequently, the samples were incubated in the dark at 37 °C for *P. aeruginosa* and at 30 °C for *P. putida*. After 60, 120, and 180 min, microbial viability was assessed using BacTiter-Glo™ reagent. The designation of live bacteria was carried out according to the procedure specified by the manufacturer (Promega Corporation, Madison, WI, USA). All experiments were conducted in three independent replicates.

### 4.4. Investigation of Protoporphyrin IX Level Variations in Bacterial Cells

Bacteria were prepared according to the protocol described in Section 4.1, with a slight modification: deionized water was substituted with a 2.5 mM solution of 5-ALA. The mixtures were incubated at 37 °C (*P. aeruginosa*) and 30 °C (*P. putida*) for 180 min (protected from light). At 15, 30, 60, 90, 120 and 180 min of incubation, the samples (250 µL) were centrifuged for 5 min at 6000 rpm. The supernatant was carefully removed, and 250 μL of lysis solution (0.1 M NaOH/1% SDS) was added to the bacterial pellet. The samples were then placed on ice for 15 min (protected from light). After this period, the samples were centrifuged again, and the fluorescence intensities of the resulting supernatants (cell-free lysates) were measured (excitation at 410 nm and emission at 631 nm).

### 4.5. Study on the Effectiveness of aPDI

The standardized suspensions of *P. aeruginosa* and *P. putida* were incubated in the dark for 120 min or 30 min, respectively, with the following treatments: (1) 5-ALA at a final concentration of 2.5 mM; (2) 5-ALA + 0.5% glucose; (3) 5-ALA + 1% glucose; (4) 5-ALA + 2% glucose. Subsequently, samples of *P. aeruginosa* were irradiated for durations of 1, 2, 3, 4, 5, 10, 15, 30, and 45 min using laser light at wavelengths of 410 nm and 635 nm. Samples of *P. putida* were irradiated under the same experimental conditions but for durations of 10, 15, 30, and 45 min. Control samples without illumination were included to assess the initial bacterial concentration. The number of viable bacteria (colony-forming units per milliliter, CFUmL^−1^) was quantified via serial dilution method. The reduction in viability of bacteria was calculated as logarithmic reduction, according to the following formula:

(1) $$R={log}_{10}N_{0}-{log}_{10}N$$

and percentage reduction, according to the following formula:

(2) $$=\frac{\left(N_{0}-N\right)\;\times \;100\%}{N_{0}}$$

where the number of bacteria before experiments is defined as $N_{0}$, and N is the number of bacteria that remained alive after irradiation time. All experiments were conducted in triplicate.

### 4.6. Research on the Mechanism of Enhancing aPDI Efficiency by Glucose

The standardized suspensions of *P. aeruginosa* and *P. putida* were incubated in the dark for 120 min or 30 min, respectively, with the following treatments: (1) 5-ALA at a final concentration of 2.5 mM; (2) 5-ALA + 0.5% glucose; (3) 5-ALA + 1% glucose; (4) 5-ALA + 2% glucose. Subsequently, all samples were centrifuged for 5 min at 6000 rpm, and the obtained supernatants were carefully removed. Lysis buffer (Tris-acetate-EDTA with 2% SDS) was added to the pellets, and all samples were placed on ice for 10 min (protected from light). After this period, the samples were centrifuged again, and the resulting supernatants (cell-free lysates) were subjected to UV-Vis spectroscopy (Shimadzu UV-1650PC), fluorescence spectroscopy, and fluorescence intensity measurements (excitation at 410 nm; emission at 631 nm) (SpectraMax Gemini spectrofluorometer, ThermoFisher Scientific). The control sample was the supernatant obtained from the lysed bacterial suspension without the addition of 5-ALA or 5-ALA + glucose, prepared according to the procedure described above.

### 4.7. Study on the Effect of Multiple aPDI Treatments on Virulence Factors

The standardized suspensions of *P. aeruginosa* and *P. putida* were incubated in the dark for 120 min and 30 min, respectively, with 5-ALA at a final concentration of 2.5 mM. Sterilized glass slides were then inoculated with 50 µL of these bacterial suspensions and irradiated for 5 min (for *P. aeruginosa*) and 45 min (for *P. putida*) using laser light at a wavelength of 405 nm. These irradiation times were selected to ensure that a sufficient number of viable cells remained in the suspensions. Following light exposure, the treated slides were immersed in 5 mL of Mueller–Hinton II broth (BTL, Łódź, Poland) and incubated at 37 °C (for *P. aeruginosa*) and 30 °C (for *P. putida*) for 24 h. After incubation, bacterial cells were harvested by centrifugation at 6000 rpm for 5 min and resuspended in buffered saline to achieve an inoculum density of approximately 1.0 × 10^−8^ CFU mL^−1^. This bacterial suspension was then used to inoculate new slides, which were subjected again to blue light irradiation for 5 min or 45 min, following the same procedure. This cycle was repeated five times. Untreated bacterial cultures served as controls. All experiments were conducted in duplicate.

The studied bacteria, *P. aeruginosa* and *P. putida* (before and after multiple light treatments), were cultured for 24 h in Tryptic Soy Broth (Sigma-Aldrich, St. Louis, MO, USA). Following incubation, the cells were centrifuged (5 min, 6000 rpm; 4 °C), and the resulting supernatants were passed through a 0.2-micrometer filter (Minisart^®^, Sartorius Stedim, Bohemia, NY, USA) and subsequently dialyzed (D-Tube Dialyzer, MWCO 3.5 kDa, Merck Millipore, Burlington, MA, USA) to remove low-molecular-weight metabolic products. These cell-free supernatants were then lyophilized at temperatures below −80 °C and under a pressure of less than 40 mTorr, and subjected to further analysis. For this purpose, 10 mg of the lyophilized supernatants was dissolved in 1 mL of deionized water. The resulting solutions were used to assess the following.

#### 4.7.1. Activity of Phospholipase C

To evaluate the activity of phospholipase C, a method based on Luberto et al. [42] was used with minor modifications. Specifically, 50 μL of p-nitrophenylphosphorylcholine (p-NPPC; 37.5 mM) (Sigma-Aldrich, USA), dissolved in 100 mM Tris-HCl buffer (pH 7.4) containing 25% glycerol, was mixed with 150 μL of cell-free supernatant from *P. aeruginosa* culture, resulting in a total reaction volume of 200 μL. The activity was assessed by measuring the increase in absorbance at 405 nm, which indicates the production of p-nitrophenol, a chromogenic compound. Absorbance was recorded every 60 s at 37 °C immediately after adding p-NPPC (Shimadzu UV-1650PC). A control sample was prepared in the same way but replaced the bacterial supernatant with distilled water. The activity of this enzyme was also measured in a cell-free supernatant obtained from a 24 h culture of *P. putida*, with the difference that the enzymatic reactions were conducted at 30 °C.

#### 4.7.2. Lipase A Activity

Lipase activity was assessed using p-nitrophenyl palmitate (p-NPP) as the substrate, following the protocol outlined by [43] with minor modifications. The assay mixture was prepared by combining 90 mL of phosphate buffer with 100 mg of gum Arabic, 207 mg of sodium deoxycholate (Sigma-Aldrich, USA), and 30 mg of p-nitrophenyl palmitate dissolved in 10 mL of isopropanol, with the pH adjusted to 8.0. To measure lipase activity, 100 μL of this assay mixture was mixed with 100 μL of cell-free filtrate from *P. aeruginosa* and incubated at 37 °C for 30 min. The reaction was then stopped by adding 0.5 mL of 3 M HCl. The mixture was centrifuged at 6000 rpm for 10 min at 4 °C, and the amount of p-nitrophenol (pNP) released was quantified spectrophotometrically at 405 nm (Shimadzu UV-1650PC). One unit of lipase activity was defined as the amount of enzyme that releases 1 μmol of pNP under these standard conditions. Lipase activity was additionally measured in a cell-free supernatant derived from a 24 h culture of *P. putida*, with the enzymatic reaction performed at 30 °C.

#### 4.7.3. Activity of Alkaline Protease

The reaction mixture consisted of 500 µL of azocasein (Sigma-Aldrich, USA) at 0.05% in 0.2 M phosphate buffer (pH 7.0) and 500 µL of the cell-free supernatant derived from the *P. aeruginosa* culture. This mixture was incubated at 37 °C for 30 min. To stop the reaction, 1 mL of 10% (*w*/*v*) trichloroacetic acid was added. After keeping the mixture on ice for 1 h, the unreacted azocasein was precipitated by centrifugation (10 min at 6000 rpm). The resulting supernatant was then combined with an equal volume of 1 N NaOH, and its absorbance was measured at 440 nm (Shimadzu UV-1650PC). A control sample was prepared identically but replacing the enzyme or bacterial supernatant with distilled water. A value of 1 enzyme activity unit (U) was defined as the amount of enzyme that produces an increase of 0.01 in absorbance at 440 nm per minute under the assay conditions. The activity of alkaline protease was also determined in a cell-free supernatant obtained from a 24 h culture of *P. putida*, with the difference that the enzymatic reaction was conducted at 30 °C.

#### 4.7.4. Elastase Activity

A volume of 1 mL of cell-free supernatant from *P. aeruginosa* was combined with 20 mg of Elastin-Congo red substrate (Sigma-Aldrich, USA) suspended in 2 mL of 0.2 M acid buffer (pH 7.4) and then incubated with shaking at 37 °C for 30 min. The reaction was stopped by adding 100 μL of 0.12 M EDTA (pH 8.0). After centrifugation (5 min; 6000 rpm; 4 °C), absorbance at 495 nm was measured using a spectrophotometer (Shimadzu UV-1650PC) calibrated with a control sample of Elastin-Congo red, which was incubated without supernatant. A value of 1 unit of elastase activity was defined as the amount of enzyme that caused an increase in absorbance at 495 nm of 0.01 after 1 min of incubation under standard assay conditions. Elastase activity was also determined in a cell-free supernatant obtained from a 24 h culture of *P. putida*, except that the enzymatic reactions were carried out at 30 °C.

All enzyme activity analyses were conducted in three independent experiments. The enzyme activity was expressed per milligram of protein present in the lyophilized supernatants. Protein concentration was determined using the Bradford assay [44].

### 4.8. Study on Pyocyanin and Pyoverdine Production

To evaluate the impact of multiple aPDI-5-ALA treatments on the production of pyocyanin and pyoverdine, a 10 μL aliquot of overnight-cultured *P. aeruginosa* was transferred to 1 mL of King’s B medium (BTL, Łódź, Poland) and incubated at 37 °C. The concentration of pyoverdine in the samples was quantified by measuring the absorbance of the cell-free supernatant at 405 nm, as described in reference [45]. The pyocyanin assay involved measuring absorbance at 520 nm in an acidic solution. Specifically, a 5 mL culture sample, grown in King’s B medium to maximize pyocyanin production, was extracted with 3 mL of chloroform, followed by re-extraction into 1 mL of 0.2 N HCl, resulting in a pink to deep red solution. The absorbance of this solution was then measured at 520 nm (Shimadzu UV-1650PC).

### 4.9. Study on Biofilm Formation

The standardized suspensions of *P. aeruginosa* and *P. putida* prepared according to the procedure described in Section 4.8 were centrifuged at 6000 rpm for 5 min. The resulting pellets were washed twice with sterile saline (0.9% NaCl) and resuspended in Mueller–Hinton Broth BTL, Łódź, Poland) to achieve an optical density at 600 nm of 1.0. A 200 µL aliquot of the bacterial suspension was then added to the wells of a sterile, black microtiter plate and incubated at 37 °C (*P. aeruginosa*) or 30 °C (*P. putida*) for 24 h to facilitate biofilm formation. Following incubation, the medium was carefully removed, and the biofilm was gently washed with sterile saline to eliminate planktonic cells. The biofilm was subsequently stained with 0.1% (*w*/*v*) crystal violet for 10 min. Excess stain was removed by rinsing with phosphate-buffered saline (PBS). The bound crystal violet was then solubilized in 5 mL of 33% glacial acetic acid, and absorbance was measured at 590 nm using a spectrophotometer. All experiments were performed in triplicate.

### 4.10. Study on Swarming Motility

Swarming motility of *P. aeruginosa* was assessed following previously established protocols with minor modifications [46]. Briefly, overnight cultures of these pathogens grown in minimal medium (62 mM potassium phosphate buffer (pH 7), 7 mM (NH_4_)_2_SO_4_, 2 mM MgSO_4_, 10 μM FeSO_4_, 0.4% (wt/vol) glucose) were point-inoculated at the center of swarm agar plates composed of 62 mM potassium phosphate buffer (pH 7), 2 mM MgSO_4_, 10 μM FeSO_4_, 0.4% [wt/vol] glucose, 0.1% [wt/vol] Casamino Acids, 0.5% [wt/vol] agar-agar. The plates were incubated upright at 37 °C and the diameter of the swarming zones was subsequently measured.

### 4.11. Fluorescence Microscopy Studies

*Pseudomonas aeruginosa* cell suspensions before and after photoinactivation with blue light in the presence of 5-ALA alone or combined with 2% glucose were stained with SYTOX^®^ Green Dead Cell Stain (ThermoFisher Scientific, Waltham, MA, USA) according to the manufacturer’s instructions. Fluorescent imaging of non-viable bacterial cells was performed using an Olympus BX60 light microscope equipped with appropriate excitation/emission filter sets (excitation bandpass filter 460–490 nm; emission long-pass filter > 515 nm).

### 4.12. Statistical Analysis

The statistical analyses of the data obtained in the experiments were performed using the STATISTICA data analysis software package (Statistica version 13.3, StatSoft Inc., Kraków, Poland) at the significance level of *p* < 0.05.

## 5. Conclusions

In conclusion, the results obtained in this study demonstrated that 5-ALA can serve as an effective prodrug in the photodynamic inactivation of *P. aeruginosa* using blue light (red light—characterized by deeper tissue penetration—can also be employed but a higher dose is necessary). The efficacy of *P. putida* cell death under light exposure did not meet expectations, indicating the need for further research in this area. A more in-depth understanding of the underlying mechanisms responsible for the reduced sensitivity of these bacteria to blue or red light appears to be necessary. It is worth emphasizing that our studies have shown that multiple exposures of *Pseudomonas* spp. to blue light did not cause increased resistance of these pathogens to aPDI. Moreover, in most cases, bacteria subjected to fivefold exposure to photodynamic inactivation exhibited higher mortality compared to those not exposed to the treatment.

## Figures and Tables

**Table 1:** Effect of multiple 5-ALA-aPDI on bacterial virulence factors (blue light irradiation).

	*P. aeruginosa* *			*P. putida* *		
	Control **	5-ALA	5-ALA + Glucose	Control **	5-ALA	5-ALA + Glucose
Alkaline protease	88.4 ± 1.7	72.8 ± 1.8	65.1 ± 2.3	92.1 ± 3.1	88.2 ± 2.9	84.4 ± 3.2
Elastase	96.2 ± 2.6	102.2 ± 2.8	93.8 ± 2.9	98.4 ± 3.1	96.1 ± 2.9	96.4 ± 3.4
Lipase A	101.2 ± 2.8	99.1 ± 3.1	97.4 ± 2.9	97.7 ± 2.4	95.1 ± 2.3	95.1 ± 1.9
Phospholipase C	105.4 ± 2.1	99.4 ± 1.6	97.0 ± 2.2	97.4 ± 3.2	96.4 ± 3.4	98.5 ± 3.3
Pyocyanin/pyoverdine production	98.4 ± 1.7	98.2 ± 2.1	101 ± 2.3	ND ***	ND	ND
Biofilm formation	90.3 ± 2.6	61.4 ± 3.3	52.4 ± 3.2	90.4 ± 3.3	78.7 ± 3.1	63.3 ± 3.1
Swarming motility	98.4 ± 2.6	80.1 ± 2.6	80.6 ± 2.8	ND	ND	ND

* The level of virulence factors detected in the cells of the tested pathogens (without 5-ALA) before irradiation was assumed as 100%. ** Controls were bacterial cells without exogenous supply of 5-ALA and/or glucose irradiated with blue light for 1 min. *** ND—not determined. Data presented as the arithmetic mean (±SD) from three independent experiments.

## Data Availability

The original contributions presented in this study are included in the article/Supplementary Materials. Further inquiries can be directed to the corresponding author.

## Supplementary Materials

The following supporting information can be downloaded at: https://www.mdpi.com/article/10.3390/ijms26157153/s1.
