# Supplementary material for: Assessing 5-Aminolevulinic Acid as a Natural Biocide Precursor for Light-Activated Eradication of Pseudomonas spp
Source: Int J Mol Sci. 2025 Jul 24;26(15):7153. doi: 10.3390/ijms26157153 (PMC12346362; doi:10.3390/ijms26157153)
Supplement: Supplementary file 1 [file ijms-26-07153-s001.zip › ijms-3707842-supplementary.pdf]

Supplementary Information

Assessing 5-aminolevulinic acid as a natural biocide precursor for light-activated eradication of *Pseudomonas* spp.

Irena Maliszewska*, Anna Zdubek

Department of Organic and Medicinal Chemistry, Faculty of Chemistry, Wrocław University of Science and Technology, Wybrzeże Wyspiańskiego 27, 50-370 Wrocław, Poland;
irena.helena.maliszewska@pwr.edu.pl; anna.zdubek@pwr.edu.pl

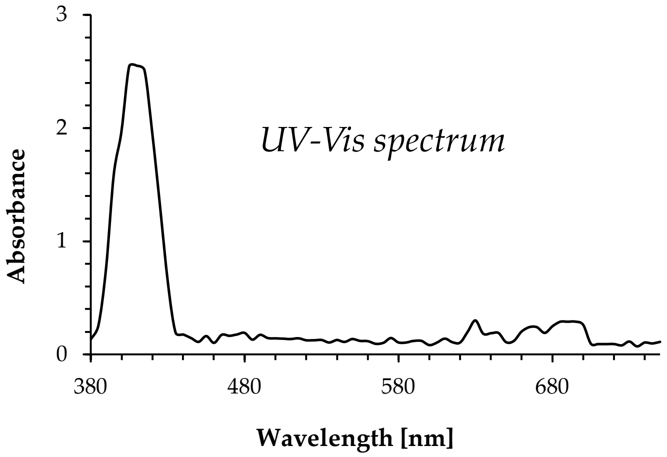

Figure S1. UV-Vis spectrum of protoporphyrin IX in lysis buffer (Tris-acetate-EDTA with 2% SDS)

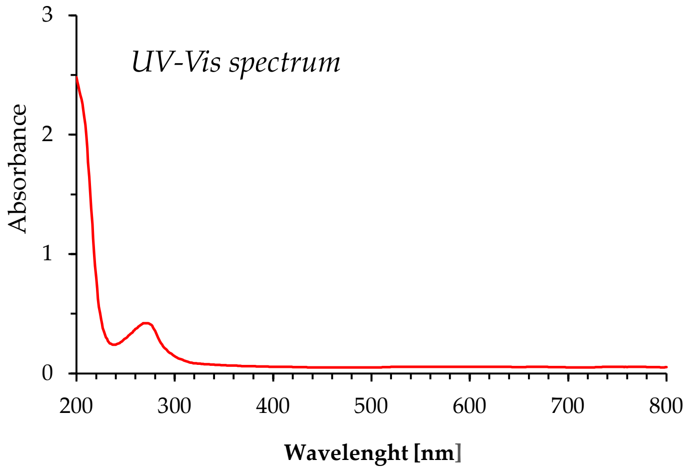

Figure S2. UV-Vis spectrum of 5-ALA in water

Table S1. Effect of multiple 5-ALA-aPDI on *P. aeruginosa* swarming zone

	Blue light (405 nm)			***Red light (635 nm)***		
	**Control***	**5-ALA**	**5-ALA+glucose**	**Control***	**5-ALA**	**5-ALA+glucose**
			**[cm]**			
**Swarming zone**	5.7±0.2	5.2±0.2	4.8±0.2	5.8±0.2	5.6±0.2	5.5±0.2

*Controls were bacterial cells without exogenous supply of 5-ALA and/or glucose irradiated with blue and red light for 10 and 45 min, rescpectively.The motality zone of *P. aeruginosa* (without 5-ALA) and before irradiation was assumed as 100% (6.0±0.2 cm). Data presented as the arithmetic mean (±SD) from three independent experiments.

Table S2. Effect of multiple 5-ALA-aPDI on bacterial virulence factors (red light irradiation; 635 nm)

	P. aeruginosa*			***P. putida****		
	Control*	5-ALA	5-ALA+glucose	Control**	5-ALA	5-ALA+glucose
**Alkaline protease**	85.9±2.1	78.4±2.3	75.4±2.6	95.4±2.8	83.3±3.2	79.8±3.5
**Elastase**	97.4±3.2	98.5±3.4	96.8±3.1	97.4±3.3	95,7±3.2	95.4±3.4
**Lipase A**	99.2±3.4	98.9±3.1	97.4±3.4	99.2±1.8	96.4±2.1	98.3±1.9
**Phospholipase C**	97.2±3.3	96.5±3.1	98.4±2.8	96.8±3.1	96.1±2.9	96.2±3.3
**Pyocyanin/pyoverdine production**	98.3±2.1	98.4±2.3	95.4±1.9	ND***	ND	ND
**Biofilm formation**	93.4±2.5	90.1±2.2	85.4±2.2	89.7±2.2	85.5±1.8	82.8±2.4
**Swarming motility**	97.4±3.2	85.8±2.9	82.3±2.9	ND	ND	ND

*The level of virulence factors detected in the cells of the tested pathogens (without 5-ALA) before irradiation was assumed as 100%. **Controls were bacterial cells without exogenous supply of 5-ALA and/or glucose irradiated with red light for 45 minutes. ***ND- not determined. Data presented as the arithmetic mean (±SD) from three independent experiments.

(a)

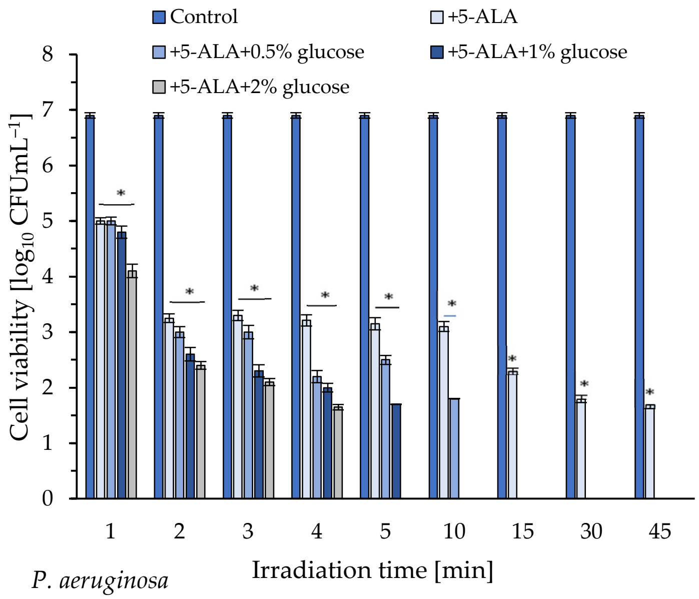
Control
+5-ALA
+5-ALA+0.5% glucose
+5-ALA+1% glucose
+5-ALA+2% glucose
Cell viability [log$_{10}$ CFUmL^{-1}]
0 1 2 3 4 5 6 7 8
1 2 3 4 5 10 15 30 45
Irradiation time [min]
P. aeruginosa

(b)

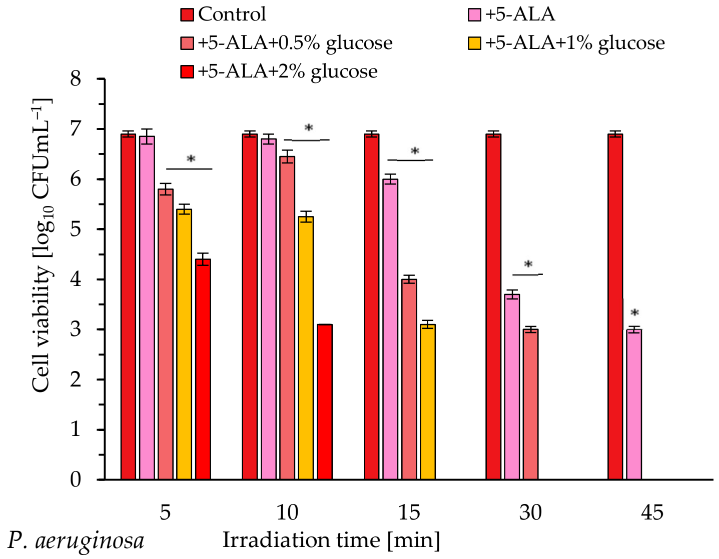
Control
+5-ALA
+5-ALA+0.5% glucose
+5-ALA+1% glucose
+5-ALA+2% glucose
Cell viability [log$_{10}$ CFUmL^{-1}]
0 1 2 3 4 5 6 7 8
5 10 15 30 45
P. aeruginosa
Irradiation time [min]

(c)

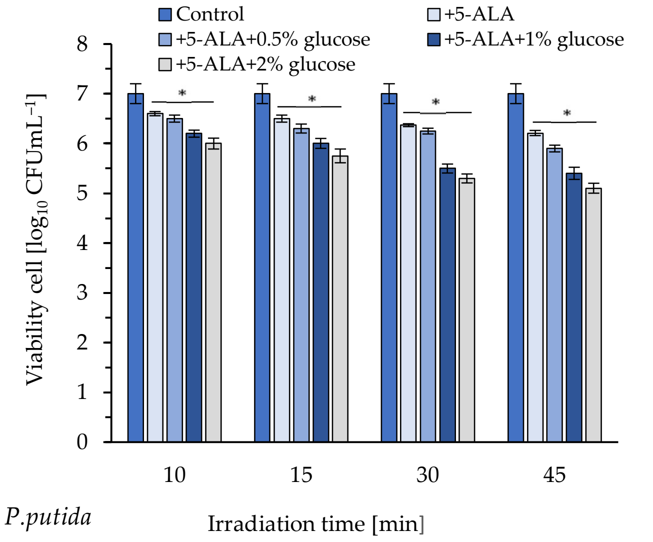
Control
+5-ALA
+5-ALA+0.5% glucose
+5-ALA+1% glucose
+5-ALA+2% glucose
Viability cell [log$_{10}$ CFUmL^{-1}]
0 1 2 3 4 5 6 7 8
10 15 30 45
P.putida
Irradiation time [min]

(d)

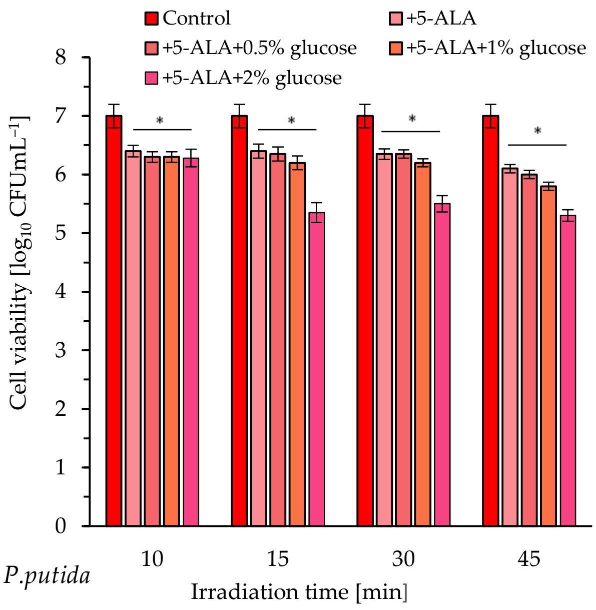

Figure S3. Efficiency of photodynamic inactivation of *P. aeruginosa* and *P. putida* under various experimental conditions. (**a**) (**c**) blue light (410 nm); (**b**) (**d**) red light (635 nm). In these experiments, cells that had previously been exposed to light five times according to the procedure described in Section 4.7. of the manuscript were used.

Data presented as the arithmetic mean (±SD) from two independent experiments. Asterisks indicate the statistical differences $p < 0.05$.
